# The cortical neurophysiological signature of amyotrophic lateral sclerosis

**DOI:** 10.1093/braincomms/fcae164

**Published:** 2024-05-13

**Authors:** Michael Trubshaw, Chetan Gohil, Katie Yoganathan, Oliver Kohl, Evan Edmond, Malcolm Proudfoot, Alexander G Thompson, Kevin Talbot, Charlotte J Stagg, Anna C Nobre, Mark Woolrich, Martin R Turner

**Affiliations:** Oxford Centre for Human Brain Activity, Wellcome Centre for Integrative Neuroimaging, University of Oxford, Oxford, OX3 7JX, UK; Nuffield Department of Clinical Neurosciences, University of Oxford, Oxford, OX3 9DU, UK; Oxford Centre for Human Brain Activity, Wellcome Centre for Integrative Neuroimaging, University of Oxford, Oxford, OX3 7JX, UK; Department of Psychiatry, University of Oxford, Oxford, OX3 7JX, UK; Oxford Centre for Human Brain Activity, Wellcome Centre for Integrative Neuroimaging, University of Oxford, Oxford, OX3 7JX, UK; Nuffield Department of Clinical Neurosciences, University of Oxford, Oxford, OX3 9DU, UK; Oxford Centre for Human Brain Activity, Wellcome Centre for Integrative Neuroimaging, University of Oxford, Oxford, OX3 7JX, UK; Department of Psychiatry, University of Oxford, Oxford, OX3 7JX, UK; Oxford Centre for Human Brain Activity, Wellcome Centre for Integrative Neuroimaging, University of Oxford, Oxford, OX3 7JX, UK; Nuffield Department of Clinical Neurosciences, University of Oxford, Oxford, OX3 9DU, UK; Nuffield Department of Clinical Neurosciences, University of Oxford, Oxford, OX3 9DU, UK; Nuffield Department of Clinical Neurosciences, University of Oxford, Oxford, OX3 9DU, UK; Nuffield Department of Clinical Neurosciences, University of Oxford, Oxford, OX3 9DU, UK; Oxford Centre for Human Brain Activity, Wellcome Centre for Integrative Neuroimaging, University of Oxford, Oxford, OX3 7JX, UK; Nuffield Department of Clinical Neurosciences, University of Oxford, Oxford, OX3 9DU, UK; Oxford Centre for Human Brain Activity, Wellcome Centre for Integrative Neuroimaging, University of Oxford, Oxford, OX3 7JX, UK; Department of Psychiatry, University of Oxford, Oxford, OX3 7JX, UK; Oxford Centre for Human Brain Activity, Wellcome Centre for Integrative Neuroimaging, University of Oxford, Oxford, OX3 7JX, UK; Department of Psychiatry, University of Oxford, Oxford, OX3 7JX, UK; Oxford Centre for Human Brain Activity, Wellcome Centre for Integrative Neuroimaging, University of Oxford, Oxford, OX3 7JX, UK; Nuffield Department of Clinical Neurosciences, University of Oxford, Oxford, OX3 9DU, UK

**Keywords:** motor neuron disease, MND, excitatory inhibitory balance, frequency band analysis, electromagnetic neuroimaging

## Abstract

The progressive loss of motor function characteristic of amyotrophic lateral sclerosis is associated with widespread cortical pathology extending beyond primary motor regions. Increasing muscle weakness reflects a dynamic, variably compensated brain network disorder. In the quest for biomarkers to accelerate therapeutic assessment, the high temporal resolution of magnetoencephalography is uniquely able to non-invasively capture micro-magnetic fields generated by neuronal activity across the entire cortex simultaneously. This study examined task-free magnetoencephalography to characterize the cortical oscillatory signature of amyotrophic lateral sclerosis for having potential as a pharmacodynamic biomarker. Eight to ten minutes of magnetoencephalography in the task-free, eyes-open state was recorded in amyotrophic lateral sclerosis (*n* = 36) and healthy age-matched controls (*n* = 51), followed by a structural MRI scan for co-registration. Extracted magnetoencephalography metrics from the delta, theta, alpha, beta, low-gamma, high-gamma frequency bands included oscillatory power (regional activity), 1/*f* exponent (complexity) and amplitude envelope correlation (connectivity). Groups were compared using a permutation-based general linear model with correction for multiple comparisons and confounders. To test whether the extracted metrics could predict disease severity, a random forest regression model was trained and evaluated using nested leave-one-out cross-validation. Amyotrophic lateral sclerosis was characterized by reduced sensorimotor beta band and increased high-gamma band power. Within the premotor cortex, increased disability was associated with a reduced 1/*f* exponent. Increased disability was more widely associated with increased global connectivity in the delta, theta and high-gamma bands. Intra-hemispherically, increased disability scores were particularly associated with increases in temporal connectivity and inter-hemispherically with increases in frontal and occipital connectivity. The random forest model achieved a coefficient of determination (*R*^2^) of 0.24. The combined reduction in cortical sensorimotor beta and rise in gamma power is compatible with the established hypothesis of loss of inhibitory, GABAergic interneuronal circuits in pathogenesis. A lower 1/*f* exponent potentially reflects a more excitable cortex and a pathology unique to amyotrophic lateral sclerosis when considered with the findings published in other neurodegenerative disorders. Power and complexity changes corroborate with the results from paired-pulse transcranial magnetic stimulation. Increased magnetoencephalography connectivity in worsening disability is thought to represent compensatory responses to a failing motor system. Restoration of cortical beta and gamma band power has significant potential to be tested in an experimental medicine setting. Magnetoencephalography-based measures have potential as sensitive outcome measures of therapeutic benefit in drug trials and may have a wider diagnostic value with further study, including as predictive markers in asymptomatic carriers of disease-causing genetic variants.

## Teaser

Trubshaw *et al*. analysed task-free magnetoencephalography in amyotrophic lateral sclerosis, identifying distinct cortical neurophysiology with biomarker potential. With increased disability, beta power was reduced in motor regions, and gamma power increased frontotemporally. Signal complexity increases may reflect changes in excitability. Global, intra- and inter-hemispheric connectivity increases likely reflect compensatory responses.

## Graphical Abstract

**Graphical Abstract:**
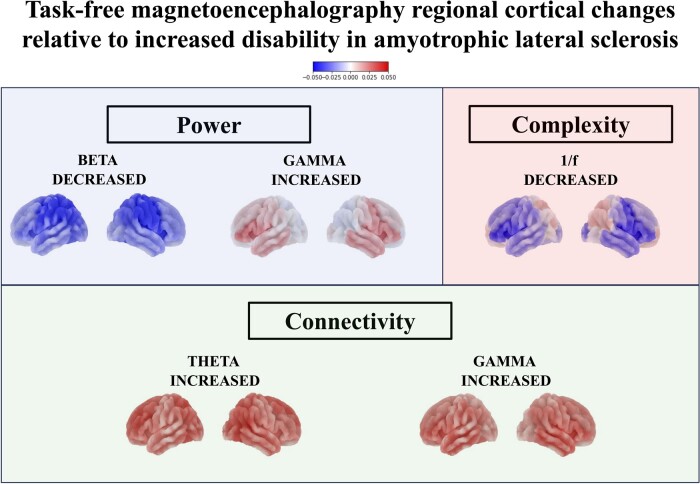

## Introduction

Amyotrophic lateral sclerosis (ALS) is an adult-onset neurodegenerative condition, defined by progressive loss of cerebral, spinal and peripheral motor system integrity. It has clinical, genetic and pathological overlap with frontotemporal dementia with recognition of a diverse set of upstream biological pathways.^1^ There are no highly effective disease-slowing drug therapies despite many trials. Such trials currently rely on insensitive outcome measures such as prolonged survival or a significantly slowed rate of disability accumulation. Biomarkers that closely reflect more individualized disease activity are needed, against which the effects of drugs can be tested more rapidly.^2^

Although much of the disability and reduced survival associated with ALS reflects peripheral neuromuscular denervation, this is associated with widespread cerebral functional changes.^3^ A consistent finding of cortical hyperexcitability in ALS using transcranial magnetic stimulation (TMS) is supported by a range of molecular, histopathological and neuroimaging evidence implicating a relative reduction of cortical inhibitory, GABAergic influences in the pathogenesis.^4,5^

Magnetoencephalography (MEG) is a neurophysiological technique that measures the oscillatory micro-magnetic fields generated primarily by cortical neuronal activity.^6^ MEG characterizes brain activity to an extremely high temporal resolution (on the millisecond level compared to seconds in functional MRI), which allows the assessment of rapid brain network dynamics, phenomena which are thought to be critical to a healthy brain function.^7^

It is possible to localize MEG brain activity to a greater degree of spatial accuracy than standard electroencephalography (EEG), and co-registering MEG signal with structural MRI further improves spatial resolution to the millimetre level allowing investigation of differences in dynamics between key brain regions.^8^ Dynamic brain activity is measured using metrics such as oscillatory power, connectivity and complexity and can be divided into canonical frequency bands, which are thought to reflect distinct neurophysiological processes.^9,10^

The clinical utility of MEG has thus far been limited to a small number of epilepsy and neurosurgery patients.^11^ The main limitations to widespread clinical MEG are its high cost, due to the need for cryogenic cooling or sensors, and complicated analysis techniques, which are inaccessible to clinicians. Both limitations are being actively addressed. The clinical utility of MEG is likely to increase in the coming years. Cost is likely to fall with the development of optically pumped magnetometers (OPMs), which currently yield similar sensitivity to standard MEG, but do not require cryogenic cooling.^12^ With the development of software libraries such as the Oxford Software Library, less technical expertise will be required to analyse and interpret MEG data.^13^

Akin to functional MRI, MEG can be acquired either during the performance of a specific task- or in a task-free, so-called resting-state. Task-MEG is used to identify the neurophysiological processes underlying specific cognitive or motor functions by instructing participants to carry out a prescribed activity, often with many repetitions. Task-free MEG provides a broader picture of whole-brain activity, quantifying interactions between oscillations in different brain regions and frequencies across large-scale functional brain networks, including the default mode, visual, temporal and sensorimotor networks.^14^ Task-free MEG is receptive to the complex and more widespread compensatory changes inherent to neurodegenerative disorders.^9^ Despite being measured at rest, these brain networks relate more broadly to functional activity and are more reliably observed in standardized way across participants than in task.^9,15^ For example, motor ability has been localized largely to beta activity in the cortical sensorimotor network and its power is reduced in motor disorders like Parkinson’s disease.^16^ Beta oscillations as measured by MEG are influenced by the activity of both cortical (mainly GABAergic inter-neuronal activity) and subcortical (basal ganglia and thalamic) structures via the thalamo–cortical loop and therefore represent a prime neurophysiological target for studying ALS.^17^

Several distinct metrics can be extracted from these MEG-based networks. Oscillatory power reflects the effective macroscopic recruitment of local neurons to cause synchronous neuronal firing in a cortical region.^18^ Motor neuronal function is known to be impaired in ALS, and therefore, oscillatory power might be expected to be altered.^19^ Cortical activity information–theoretic complexity, which is reduced in Parkinson’s disease, describes irregularity of activity and likely reflects local cortical micro-pathologies, exhibited as neurophysiological disorganization.^16,20^ The 1/*f* exponent is one such composite measure, which describes the slope of the power spectrum and is a broad measure theorized to capture cortical excitability and signal complexity.^21-23^ Lower 1/*f* (shallower slope) is associated with greater complexity, which is thought to reflect higher cortical excitability.^22^ For a condition like ALS, in which cortical hyperexcitability has been a consistent observation, 1/*f* might be expected to be reduced. Connectivity, of which amplitude envelope correlation is one metric, can be thought of as the degree of synchronous activity between separate brain regions and is therefore a measure of effective long-range communication.^15^ Connectivity in ALS might be expected to increase or decrease depending on whether the changes are compensatory or reflect neuronal loss, respectively.^24^

Only a small number of resting-state MEG and EEG studies examining ALS exist in the literature. Amongst these, results are highly heterogeneous with conflicting findings in oscillatory power and connectivity, with complexity being largely unexamined.^19,24-29^ This heterogeneity in findings might be explained by variability in methods. We sought to develop an easily reproducible method to characterize the cortical neurophysiological signature in terms of MEG power, complexity and connectivity, with the aim of identifying candidate pharmacodynamic biomarkers to accelerate therapeutic discovery.

From these neurophysiological measures, we aimed to build a single predictive model using machine learning techniques, to test assess whether these MEG measures could be useful, generalizable and predictive biomarkers in ALS.

## Materials and methods

### Participants

This was an observational, cross-sectional, case–control study of task-free MEG. Referrals to the Oxford Motor Neuron Disease Care and Research Centre with a diagnosis of apparently sporadic ALS as defined by the Gold Coast criteria were eligible for study.^30^ Those with a family history of ALS or any pathological genetic variant detected through routine clinical testing were excluded from the analysis presented here. The revised ALS Functional Rating Scale (ALSFRS-R, 0–48, where lower score reflects greater disability) and a clinical upper motor neuron (UMN) score based on pathological reflexes (see Menke *et al.*^31^) were noted at the time of study.^32^ Healthy controls, comprising mainly spouses and friends of the patients, were matched for age and sex, with no significant medical history. All participants provided informed, written consent. The study was approved by the National Research Ethics Service Committees (14/SC/0083, 17/SC/0277).

### MEG acquisition

Participants underwent MEG for eight to ten continuous minutes. Participants were instructed to remain still, visually fixating on a cross displayed 120 cm in front of them. Prior to MEG acquisition, a Polhemus 3D tracking system was used to record each participant’s head shape relative to three fiducial points, located on the nasion and preauricular landmarks. Five head position indicator (HPI) coils were fixed to the participant’s nasion and bilateral supra-orbital and posterior auricular regions. The locations of HPI coils and three anatomical fiducials were digitized using the tracking system (Polhemus, EastTrach 3D) to define the subject-specific Cartesian head coordinate system. These coils were localized within a scanner space continuously throughout MEG data acquisition. Participants underwent a T_1_-weighted structural MRI scan with a Siemens Trio 3T (settings: 3-dimensional, whole-brain, magnetization-prepared rapid-acquisition gradient echo sequence, repetition time = 1900 ms, echo time = 4.7 ms, flip angle 8°, 1 mm isotropic resolution, 7 min acquisition time) within 1 month for MEG co-registration.

### Data processing

The OHBA Software Library (OSL) v0.5.1 Python package was used for data processing.^13^ For pre-processing, the MaxFilter software version 2.2 was used to remove noise, detect bad channels and correct for head movement through the temporal signal space separation algorithm (tSSS).^33^ A bandpass filter was employed between 0.5 and 125 Hz, a notch filter at 50 and 100 Hz, resampled to 250 Hz with automated bad segment removal, and a final bandpass filter was applied before beamforming between 1 and 80 Hz.^34^ Co-registration, forward modelling and beamforming were carried out with OSL’s RHINO tool. Co-registration made use of each subject’s structural MRI, three fiducials and >100 head-shape points located on the scalp. Forward modelling used a single layer representing the inner skull surface. Beamforming to a regular 8 × 8 × 8 mm dipole grid was carried out using a data covariance matrix regularized to have a rank of 60, with dipole orientations obtained by maximizing each dipole’s power.^35^

The data was then parcellated into regions of interest using a reduced parcellation adapted from Glasser *et al*.^36^ (see Supplementary Table 1). Parcellation was carried out using the ‘spatial basis’ method in OSL’s RHINO, where a parcel time course was taken to be the first principal component from all voxels, weighted by the spatial map for that parcel.^37^ Finally, symmetric multivariate leakage reduction was carried out to reduce spatial leakage between parcels, including so-called ‘ghost-interactions’.^37^

To measure static oscillatory power, the power spectral densities (PSDs) were calculated using Welch’s method with a Hann window of 2 s length from each standardized (*z*-transformed) parcel time course.^38^ Power was estimated across six canonical frequency bands: delta (1–4 Hz), theta (4–7 Hz), alpha (7–13 Hz), beta (13–30), low-gamma (30–48) and high-gamma (52–80 Hz). The FOOOF algorithm (version 1.0.0) was used to parameterize PSDs between 1 and 70 Hz and extract the aperiodic component (a description of the general slope of the PSDs across the entire frequency range) at each parcel as a measure of complexity. Settings included peak width limit: 0.5–12.0, maximum number of peaks: ∞, minimum peak height: 0.05, peak threshold: 2.0 and aperiodic mode: fixed.^21^ As a measure of connectivity, amplitude envelope correlations (AEC) were calculated for each of the six frequency bands separately from the Hilbert transformed standardized (*z*-transformed) parcel time courses.^37^ From the AEC matrices, the mean values of the connections between each region and all other regions, each region and all other regions in the contralateral hemisphere and each region and all regions in the ipsilateral hemisphere were taken, to construct metrics for global, inter- and intra-hemispheric connectivity, respectively.

### Statistical analysis

Python (version 3.8.15), GLMTools (version 0.2.0) and Scipy (version 1.10.0) packages were used for all statistical analyses.^39,40^ Each metric of cortical activity (static power, 1/*f* exponent, AEC) was summarized using a separate general linear model (GLM), including confound regressors for age, sex and handedness. To assess group differences between ALS patients and HCs, we included two separate regressors for each group and used the two-tailed contrast shown in Supplementary Fig. 1 (C1: group). The null hypothesis stated no group differences (ALS = HC), whilst the alternative hypothesis posited ALS≠HC. Statistical significance was determined using non-parametric permutation testing.^41^ Multiple comparisons (due to fitting a separate GLM to each parcel and frequency) were accounted for by using the maximum *t*-statistic method. Test statistics were calculated on the true dataset and then for 5000 random permutations of that data (assignment to groups). By taking the maximum test statistic across all parcels and frequencies in each permutation, a null distribution was created, against which the *t*-statistics from the true dataset were compared. *P*-values for each frequency band and parcel were derived by calculating the proportion of maximum *t*-statistics from permutations, which were smaller than the observed statistic from the actual data. For example, if the observed *t*-statistic was more extreme than 95% of the null distribution of maximum *t*-statistics, then *P* = 0.05. *P* < 0.05 was considered significant.^42^ This non-parametric statistical method controls the family-wise error rate and makes no assumptions on the distribution of the data. We report the *t*-statistic using the notation t(DOF) where DOF is the degrees of freedom. To compare patients and controls whilst allowing for differences in clinical score in the patients, the relationships between UMN score and change-from-baseline ALSFRS-R and cortical activity, respectively, were explored by replacing the ALS and HC regressors with each score (see Supplementary Fig. 2 for the design matrix and contrast used for statistics significance testing). Healthy controls were assigned a ‘healthy’ clinical score of 0. A supplementary analysis examined progression rate of ALSFRS-R [*δ*ALSFRS-R = (48-ALSFRS-R)/time from symptom onset].

### Model training and evaluation

To test the utility of MEG-extracted features (power, AEC and 1/*f* exponent) as predictive biomarkers of disease, a random forest regression model (scikit learn’s RandomForestRegessor) was trained on the MEG-extracted features from ALS patients to predict change-from-baseline ALSFRS scores.^43^ This is a non-parametric method that allows for non-linear associations between features (extracted from the MEG data) and target (ALSFRS scores). Random forest models build multiple decision trees and average their predictions, making them robust to overfitting and adaptable to complex relationships without assumptions such as linearity.^44^

The input features included the AEC values of each pairwise connection and each parcel’s power value per-frequency band (six canonical frequency bands). The 1/*f* exponent value from each parcel was also included.^43^

The random forest regressor was trained and validated using the nested leave-one-out cross-validation (LOOCV) approach. Each iteration of the model was optimized by using stepwise feature selection, choosing the features, which produced the highest *k*-fold cross-validated *R*^2^ in the training data for that model (*k* = 5). The left-out (unseen) ALSFRS value was then predicted. Predicted ALSFRS values from the models were gathered and compared against the corresponding true ALSFRS values using linear regression. The *R*^2^ and *P*-value were reported.

## Results

### Demographics

Of the recruited participants (*n* = 92), four were excluded for missing scan data due to intolerability to MRI, missed appointment or cancellations during COVID pandemic. One ALS participant was excluded due to a visually noisy and uninterpretable MEG time course. There were no statistical differences between the healthy controls and patients in terms of age, sex or handedness (**Table 1**).

**Table 1:** Participant details

Group	*n*	Mean age (SD)	% male	% right-handed	Mean ALSFRS-R (SD)	Mean UMN score (SD)	ALSFRS-R rate of decline (change in ALSFRS/years from onset)	Time from disease onset to study (years)	Location of disease onset limb/bulbar/neck %
ALS	36	62 (10)	72	88	37.6 (7.0)	9.0 (4.4)	7.2 (6.8)	2.1 (1.3)	77/21/2
Controls	51	60 (13)	57	94	NA	NA	NA	NA	NA
Total	87	62 (11)	65	91	43.8 (6.8)	3.7 (5.3)	NA	NA	NA

### MEG measures of brain dynamics

#### Power

**Figure 1:**
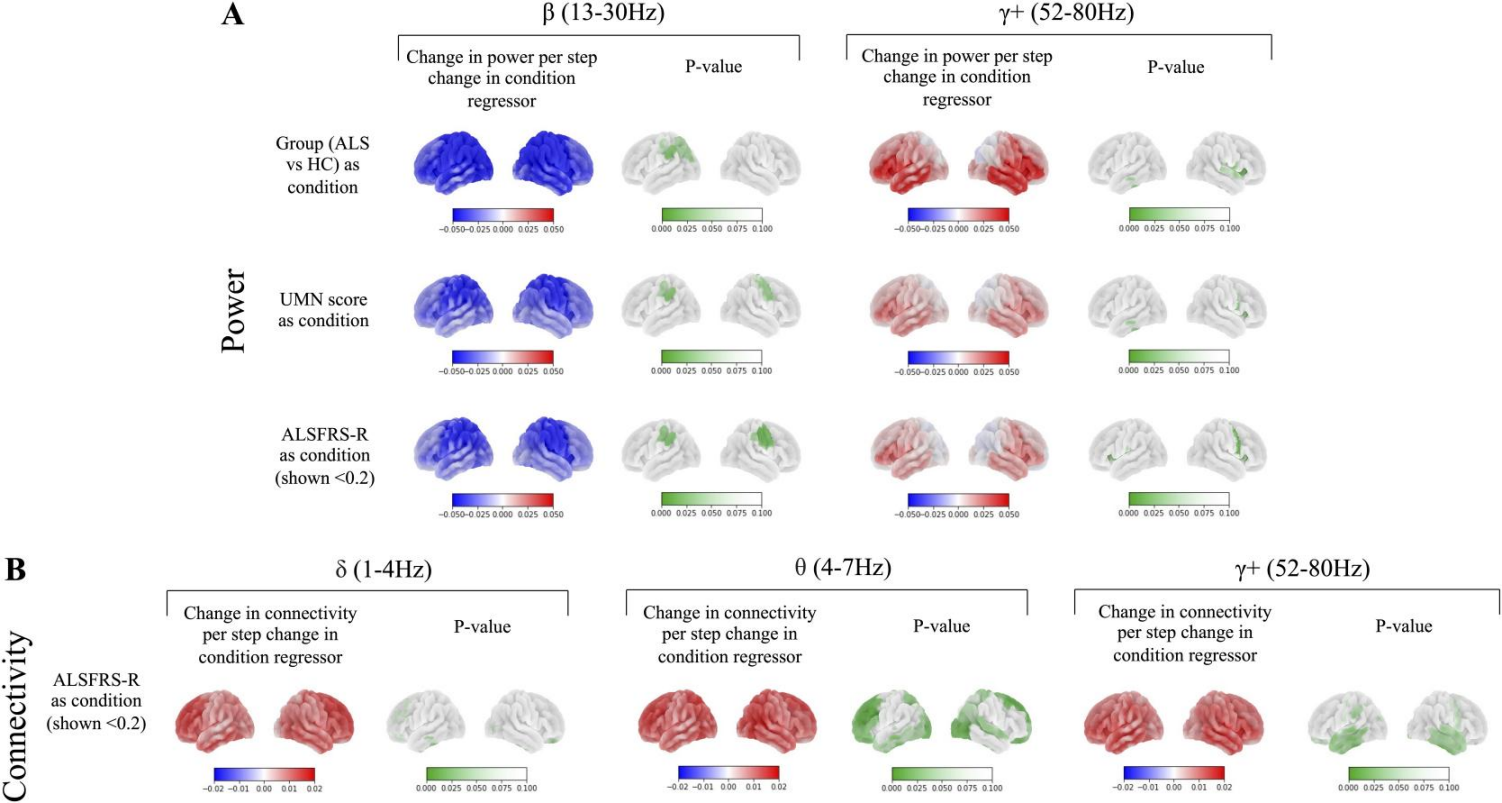
**Power and connectivity heatmaps.** This shows the effect of group [amyotrophic lateral sclerosis (ALS, *n* = 36)—healthy controls (HC, *n* = 51)], upper motor neuron (UMN) score and functional rating score (ALSFRS-R) on (**A**) power and (**B**) global connectivity. The *P*-value maps show regions, in which a step change in the respective regressor significantly explained differences in power or connectivity (amplitude envelope correlation, AEC) in that region (see Supplementary Fig. 1  and 2 for further explanation of statistical design). Significance was assessed using a permutation-based general linear model, correcting for multiple comparisons across frequencies and regions. Worsening (a larger fall from baseline) ALSFRS-R explained a larger area of sensorimotor beta power decrease [*t*(77) = 4.357, *P* = 0.010] and gamma increase [*t*(77) = 3.996, *P* = 0.015] compared to both the UMN score and group regressors. Worsening ALSFRS-R also explained areas of increased right medial temporal delta connectivity [*t*(77) = 3.875, *P* = 0.026], frontal [*t*(77) = 4.393, *P* = 0.008] and occipital [*t*(77) = 3.741, *P* = 0.033] theta connectivity and temporal gamma connectivity [*t*(77) = 3.380, *P* = 0.067].

The ALS group showed decreased oscillatory power in the beta band in the left inferior parietal cortex parcel compared to controls [*t*(77) = 3.729, *P* = 0.035] (Fig. 1A**—**left). The areas of significantly lower beta power in sensorimotor regions widened to include the right inferior parietal cortex parcel when higher UMN score was used to predict power [*t*(77) = 3.752, *P* = 0.038]. When lower ALSFRS-R was used to predict power, there was also a significant decrease in beta in the right inferior parietal cortex [*t*(77) = 3.882, *P* = 0.036], inferior somatosensory and motor cortex parcels [right: (*t*(77) = 4.357, *P* = 0.010], left: [*t*(77) = 3.736, *P* = 0.049)]. High-gamma frequency power was increased in the ALS group in the right insular and frontoparietal operculum parcel [*t*(77) = 3.996, *P* = 0.015] (Fig. 1A**—**right). Switching the regressor to ALSFRS-R broadened the area of significant difference to include the right premotor cortex [*t*(77) = 4.472, *P* = 0.007] and bilateral insular and frontoparietal operculum parcels [*t*(77) = 4.319, *P* = 0.011], [*t*(77) = 3.751, *P* = 0.047] for right and left parcels, respectively. There was no significant effect of group, ALSFRS-R, *δ*ALSFRS-R or UMN score in other frequency bands.

#### Complexity

**Figure 2:**
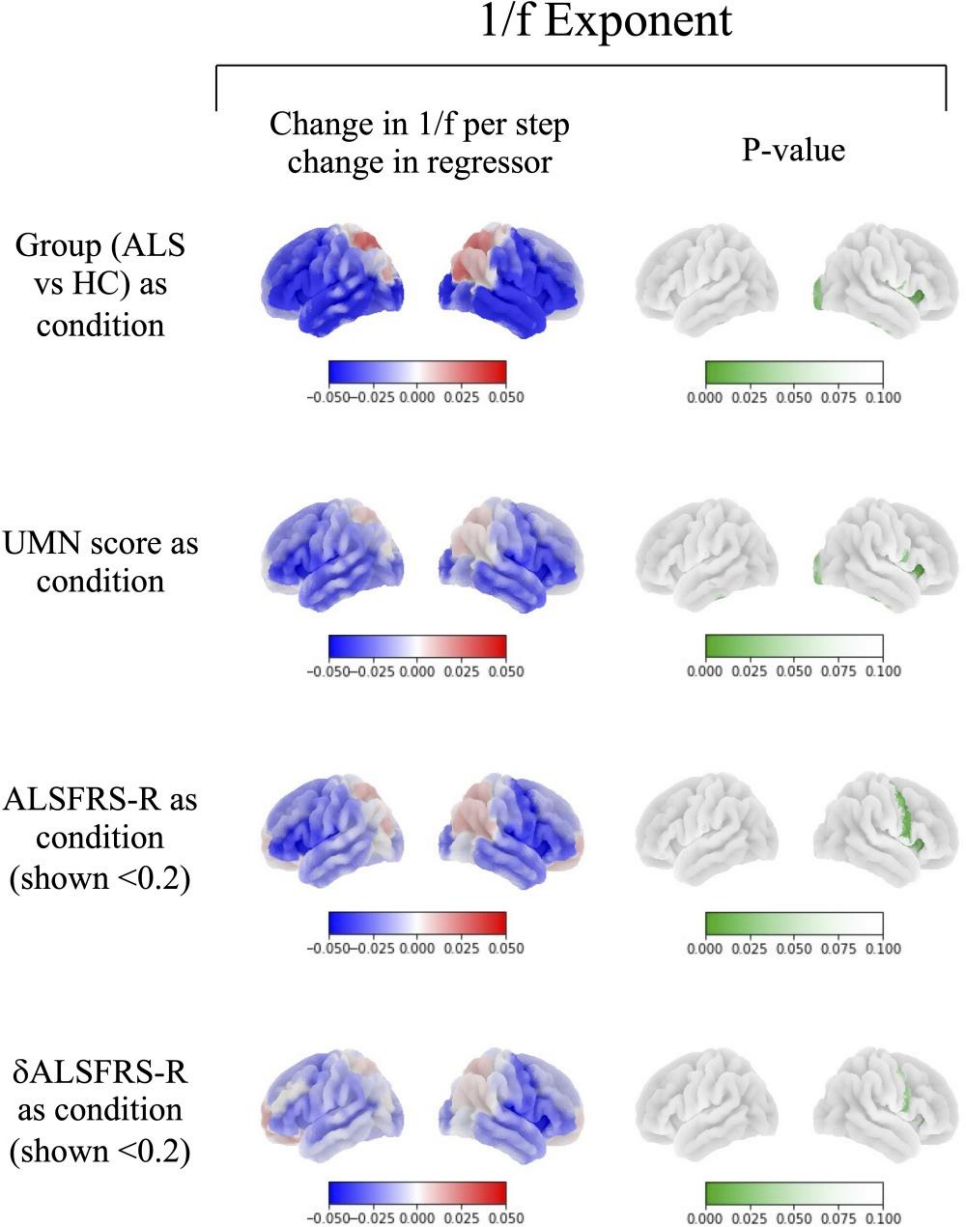
**Complexity.** This shows changes in 1/*f* exponent as predicted by group, [amyotrophic lateral sclerosis (ALS, *n* = 36)—healthy controls (HC, *n* = 51)], upper motor neuron (UMN) score, functional rating score (ALSFRS-R) and disability progression rate (*δ*-ALSFRS-R). Significance was assessed using a permutation-based general linear model, correcting for multiple comparisons across regions. ALSFRS-R and *δ*-ALSFRS-R significantly predict decreased 1/*f* in right premotor regions [(*t*(77) = 4.522, *P* = 0.007] and [*t*(77) = 3.532, *P* = 0.024), respectively], whilst group and UMN score significantly predict the reduction in the right fronto–parietal operculum [*t*(77) = 4.150, *P* = 0.003] (see Supplementary Fig. 2 for further explanation of statistical design).

Analysis of 1/*f* showed a significantly lower (flatter) exponent, which is likely related to a higher complexity of brain activity in ALS centred around the right frontoparietal operculum [*t*(77) = 3.840, *P* = 0.010] (Fig. 2). When UMN score was used as the regressor, this area was unchanged [*t*(77) = 4.150, *P* = 0.003]. With ALSFRS-R as the regressor, the area of significance expanded to include the right premotor strip [*t*(77) = 4.522, *P* = 0.007]. Furthermore, higher *δ*ALSFRS-R (higher disability progression rate) significantly explained decreases in 1/*f* in the right frontoparietal operculum [*t*(77) = 3.532, *P* = 0.024].

#### Connectivity

No significant connectivity differences were seen when the ALS and control groups were compared. However, in relation to increased disability in the ALS group, areas of increased global connectivity in the theta band were seen in frontal [(*t*(77) = 4.393, *P* = 0.008), (*t*(77) = 3.990, *P* = 0.021) for right and left dorsolateral prefrontal cortex parcels, respectively], occipital [(*t*(77) = 3.741, *P* = 0.033), (*t*(77) = 3.772, *P* = 0.032) for right and left primary visual parcels, respectively] and superior parietal parcels, including sections of the right supplementary and cingulate motor areas [(*t*(77) = 3.668, *P* = 0.038), (*t*(77) = 3.533, *P* = 0.050), respectively] (Fig. 1B**—**left). Global connectivity in the delta band was also increased significantly with greater disability in the right medial temporal cortex [*t*(77) = 3.875, *P* = 0.026] (Fig. 1B**—**middle). Although not statistically significant, temporal connectivity in the high-gamma band was increased in relation to greater disability [(*t*(77) = 3.380, *P* = 0.067), (*t*(77) = 3.473, *P* = 0.057) for right and left lateral temporal cortex parcels, respectively] (Fig. 1B**—**right).

**Figure 3:**
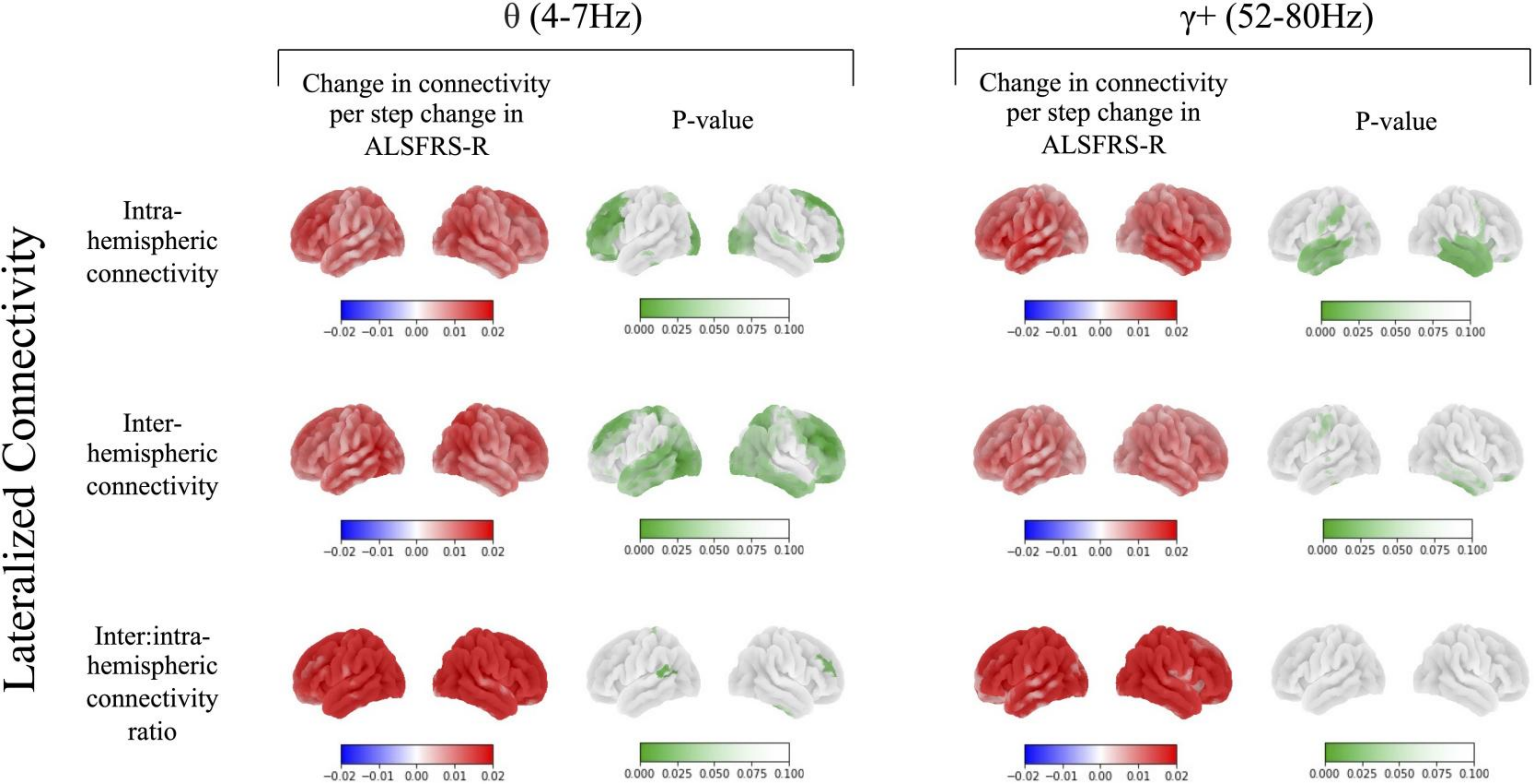
**The lateralization of connectivity.** This shows changes in lateralized connectivity associated with worsening functional rating scores (ALSFRS-R, *n* = 87, see Supplementary Fig. 2 for further explanation of statistical design). Significance was assessed using a permutation-based general linear model, correcting for multiple comparisons across frequencies and regions. The increased frontal theta connectivity seen with worsening ALS disability is comprised of increases in both inter- [*t*(77) = 3.657, *P* = 0.042] and intra-hemispheric [*t*(77) = 4.255, *P* = 0.0128] connectivity (amplitude envelope correlation, AEC). Frontal inter-hemispheric changes are significantly higher than intra-hemispheric [*t*(77) = 4.693, *P* = 0.005]. The increase in temporal gamma connectivity with worsening ALSFRS-R is driven by higher intra-hemispheric, rather than inter-hemispheric connectivity [*t*(77) = 3.881, *P* = 0.029].

The increases in temporal high-gamma (Fig. 3**—**right) and delta seen in the global connectivity analysis with greater disability were driven by intra-hemispheric rather than inter-hemispheric changes [right lateral temporal cortex parcel intra-hemispheric gamma: *t*(77) = 3.881, *P* = 0.029; right medial temporal cortex parcel intra-hemispheric delta: *t*(77) = 4.115, *P* = 0.018]. Conversely, the theta changes in global frontal, occipital and motor connectivity were driven by inter-hemispheric rather than intra-hemispheric connectivity (Fig. 3**—**left**)** [left dorsolateral prefrontal cortex parcel inter-hemispheric theta: *t*(77) = 4.693, *P* = 0.005; left visual cortex parcel inter-hemispheric theta: *t*(77) = 4.326, *P* = 0.012; right cingulate motor area parcel inter-hemispheric theta: *t*(77) = 3.731, *P* = 0.038]. The superior dorsolateral prefrontal cortex was the only region, which was significantly hyperconnected both intra-hemispherically [*t*(77) = 4.255, *P* = 0.0128, *t*(77) = 3.898, *P* = 0.028, for right and left, respectively] and inter-hemispherically [*t*(77) = 3.657, *P* = 0.042, *t*(77) = 3.962, *P* = 0.023, for right and left, respectively]. The left dorsolateral prefrontal cortex and right temporo–parieto–occipital junction were relatively more connected to contralateral regions than to ipsilateral regions in relation to greater disability [*t*(77) = 4.018, *P* = 0.032, *t*(77) = 4.522, *P* = 0.007, respectively]. There were no significant changes detected in the remaining frequency bands.

All significant results from individual regions can be found in Supplementary Table 2.

**Figure 4:**
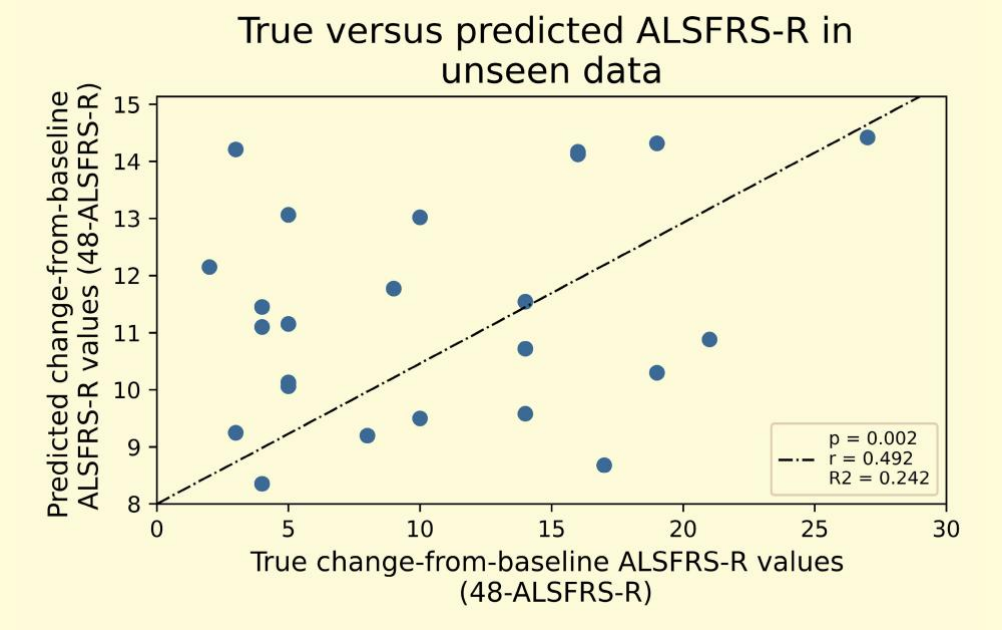
**Random forest regression model.** This shows the results of true versus predicted change-from-baseline functional rating score (48-ALSFRS-R) values in unseen data from a nested leave-one-out cross-validation random forest regression model. Only amyotrophic lateral sclerosis (ALS) patients were included (*n* = 36). The model was trained using all available magnetoencephalography metrics (power, complexity and connectivity). Each point on the graph represents each ALS patient’s true versus predicted change-from-baseline ALSFRS-R score, estimated from the model. Linear regression was then used to evaluate the relationship between true and predicted change-from-baseline ALSFRS-R values.

Random forest regression was able to significantly predict change-from-baseline ALSFRS-*R* scores from AEC, power and 1/*f* (*R*^2^ = 0.242, *P* = 0.002) (Fig. 4**)**.

## Discussion

This study established the utility of MEG for deriving network-level cortical neurophysiology biomarkers in ALS, with disability-related changes noted in oscillatory power, complexity and connectivity. Published studies of task-free MEG in ALS have described global increases in connectivity or localized increases in the extreme low and high frequencies (delta and gamma), involving fronto–temporal, sensorimotor and visual networks.^19,25-27,45^ Power-related findings in ALS have been more heterogeneous, likely because standard methods to derive and standardize MEG power spectra have not been consistently employed.^24,28,29,46^

### The power signature

Reduced beta and increased gamma frequency power focused within the sensorimotor cortex appears to be a key feature of the motor cortical lesion in ALS, consistent amongst otherwise heterogeneous observations of published studies.^19,25,47,48^ The decrease in sensorimotor beta power observed is considered to potentially reflect dysfunction of inhibitory interneurons, which may underpin the changes in complexity and connectivity.^49^ It is established that intrinsic inhibitory GABAergic modulation via interneurons is essential for controlling movement in the healthy brain.^50^ Administering a GABA_A_ agonist to healthy participants increased MEG beta power in the motor cortex.^49^ Another study found that GABA_A_- and gap junction-blockers reduced beta oscillation amplitude.^51^ GABA_A_ receptor densities were negatively correlated with cortical gamma power.^52^ Cortical beta oscillations are also known to be reduced in conditions, which damage the inhibitory projections from the basal ganglia and thalamus, as seen in stroke and Parkinson’s disease.^17^

Cortical inhibitory influences have been demonstrated as dysfunctional in ALS using a range of tools.^5^ The ‘boundary shift’ effect in cerebral activity during a motor task using positron emission tomography (PET) was suggested to reflect loss of local inter-neuronal circuit control, with later ligand PET studies showing reduced regional cortical GABA_A_ receptor binding in ALS.^53,54^ Promoting the activity of layer V interneurons delayed the onset of associated deficits and prolonged survival in an ALS mouse model.^55^ The observation that both considerations of the range of both disability and UMN score within the participant group produced similar results supports the view that inter-neuronal dysfunction is common across at least some of the phenotype heterogeneity. The fact that rate of disability progression did not significantly influence this metric may indicate that MEG power reflects the extent of cortical damage rather than disease activity.

### Complexity

The FOOOF method splits the PSD into aperiodic (1/*f*-like shape) and periodic components and claims to represent the intrinsic excitatory:inhibitory balance and oscillatory activity of the cortex, respectively.^21^ The splitting of the PSD into two components is a relatively novel approach, and therefore, its utility and meaning are pending full evaluation.^21^ A flatter aperiodic 1/*f* slope is likely to be related to increased complexity of the MEG signal as this represents a higher low:high-frequency power ratio. It is likely however that the aperiodic 1/*f* slope, as a broad-spectrum measure, is influenced by many more factors than just signal complexity.^22,23^ The 1/*f* has previously been specifically related to the excitatory–inhibitory balance of cortical activity and investigated in conditions such as Parkinson’s disease, fragile X and cervical dystonia.^20,22^ Although the relationship between 1/*f*, complexity and excitatory activity has not been conclusively proven, the finding that the premotor cortex showed a lower 1/*f* exponent (flatter curve) associated with higher *δ*ALSFRS-R suggests regional hyperexcitability in relation to more aggressive rates of disability progression ALS. Future work is needed to explore how the 1/*f* metric is related to TMS-based measures of cortical excitability.

### Connectivity

Though some studies have noted general increases in connectivity metrics in ALS, this analysis found no significant group–level difference in connectivity between groups, perhaps due to the very stringent multiple comparisons correction employed.^19,24,25^ ALSFRS-R regression was associated with widespread areas of increased connectivity in the theta and gamma bands in relation to increased disability. This is presumed to most likely reflect compensatory activity, though a study of MRI-based structural and functional connectivity in relation to rate of disability progression has provided some support for the increase being potentially linked more directly to pathogenesis.^56^ The observation of increased intra-hemispheric lateralization of gamma connectivity and of increased both intra- and inter-hemispheric lateralization of theta activity corroborates the findings of a published study in ALS.^29^ Through dynamic analyses of healthy brain activity using a hidden Markov model approach, temporal activity is known to be unilateral whilst frontal, default mode and visual networks are bilateral.^57^ Thus, ALS patients appear to show an exaggeration of normal physiological, with hyper-lateralized temporal gamma activity and hyper-generalized low-frequency connectivity in frontal and occipital regions. Sensorimotor connectivity remained largely unchanged. This provides further evidence that these changes in connectivity are likely to be compensatory.

The particular increase in inter-:intra-hemispheric connectivity observed in the dorsolateral prefrontal cortex and the temporo–parieto–occipital junction, two areas, which are integral to cognitive processing, might reflect the corpus callosum disruption known to be a consistent feature of ALS pathology.^58,59^ The dorsolateral prefrontal cortex was the only region, which was hyperconnected in all three possible connectivity measures (global, intra- and inter-hemispheric), which supports the other neuroimaging and cognitive studies specifically identifying this region in the wider cognitive effects of ALS pathology.^60-63^

### The potential for MEG as a predictive biomarker

Machine learning models have been previously used to predict disease severity from MEG in ALS, most notably by Polverino *et al*. who calculated a per-frequency band ‘functional repertoire’ score, a measure of brain network flexibility, and used it to predict ALSFRS.^64^ A key advantage to using machine learning techniques is their ability to show model generalizability to new, unseen data.

We used the random forest method, which allows for non-linear relationships between features and outcomes, is able to reduce noise by selecting important features to include in the model through cross-validation on the training set and reduces overfitting as the output is based on decision tree majority voting.^44^ The *R*^2^ of our model was 0.24 showing that our MEG-based measures are able to explain a significant proportion of the variance in disability scores. It has been proposed that brain network dynamics are fundamental to healthy brain function.^65^ Future iterations of this model might seek to include brain dynamics combined with important static features in larger, unseen datasets.

### Insights from other neurodegenerative diseases and aging

Neurodegenerative diseases are often framed simplistically as accelerated aging processes. Premorbid structural and functional MRI networks have been hypothesized to define some of the phenotypes associated with neurogenerative disorders.^66^ The most consistent finding in MEG studies of aging is of increased motor beta power, the opposite from findings in all three major neurodegenerative diseases: ALS, Parkinson’s and Alzheimer’s disease.^16,67,68^ This might support the view of it being the signature of compensatory responses more generically. It raises the tantalizing longer-term prospect of using cortical activity measures in population screening for early signs of ‘deviation’ from healthy aging that reflect sub-clinical pathology and when intervention might be much more effective. Gamma power, in contrast to the ALS findings, has been found to be reduced in Parkinson’s disease and Alzheimer’s disease.^19^ Furthermore, metrics of MEG signal complexity in Parkinson’s disease and Alzheimer’s disease have invariably been reported as reduced compared to the strong increases observed in ALS, which might provide some specificity to support the longer-term aspirations for asymptomatic population screening.^16,67^

### Limitations

The GLM and choice of the conservative maximum statistic correction mitigated false positive (type I) errors despite the relatively small sample size. We chose not to use cluster-based correction due to the higher risk of type 1 error with this method.^69^ Bonferroni correction is regarded as a highly conservative correction method, which excessively reduces power.^70^ Although no cases meeting diagnostic criteria for frontotemporal dementia were included in this study, the cortical changes in relation to more subtle changes in cognition and behaviour associated with ALS pathology will require more dedicated study. Our study did not examine the dynamic interactions of brain regions on a ‘state-level’ that has recently been achieved and needs future consideration in longitudinal studies.^71^

### Concluding remarks

The cortical neurophysiological signature of ALS is one of reduced beta and increased gamma power, hypothesized to reflect reduced inhibitory interneuronal influences, supported by the 1/*f* exponent findings. Increased complexity more broadly might reflect a disordered, hyper-excitable cortex, with increased connectivity measures reflecting compensatory processes that, pending further studies, might influence prognostic variation and other phenotypic heterogeneity. MEG has a potential as a predictive biomarker for ALS, and improvements to models may involve advanced dynamic analyses. Future longitudinal studies should include asymptomatic carriers of the commonest hereditary causes of ALS to try to unravel the evolution of any such compensatory cortical responses and markers of presymptomatic phenoconversion.

## Supplementary Material

fcae164_Supplementary_Data

## Data Availability

Data are not publicly available as they contain patient-sensitive information. Requests for access will be considered on submission to the authors. Example code for data processing is available at: https://github.com/OHBA-analysis/osl-dynamics/tree/main/examples/static.
