# Supplementary material for: The cortical neurophysiological signature of amyotrophic lateral sclerosis
Source: Brain Commun. 2024 May 13;6(3):fcae164. doi: 10.1093/braincomms/fcae164 (PMC11109820; doi:10.1093/braincomms/fcae164)
Supplement: fcae164_Supplementary_Data [file fcae164_supplementary_data.pdf]

Supplementary material

Parcellation method

The data was divided into 52 parcels using a parcellation created by combining structural information from the Human Connectome Project Multimodal Parcellation (HCP-MMP) with functional information of lead field data obtained from a previous dataset of healthy controls and Parkinson's disease patients.[1] Following the procedure described by Tait *et al.* the optimal number of parcels per HCP-MMP region was calculated by normalizing each region's importance and multiplying it by the desired number of parcels.[2] The splitting of regions was achieved by merging single regions of interest within a region, based on information provided in the Supplementary Information of Glasser et al.[1] To obtain a fine-grained description of the somatosensory area the parcels containing primary motor and sensory cortex were subdivided into two sub-parcels based on K-means clustering of voxel coordinates. Overall, this resulted in a reduced version of the HCP-MMP atlas with 52 parcels, the MNE co-ordinates of which can be found in **Supplementary Table 1**.

General linear model design

See **Supplementary Figure 1** and **Supplementary Figure 2**

Table of results

See **Supplementary Table 2**

Supplementary references

1. Glasser MF, Coalson TS, Robinson EC, et al. A multi-modal parcellation of human cerebral cortex. *Nature*. 2016;536(7615):171-178. doi:10.1038/nature18933

2. Tait L, Özkan A, Szul MJ, Zhang J. A systematic evaluation of source reconstruction of resting MEG of the human brain with a new high-resolution atlas: Performance, precision, and parcellation. *Human Brain Mapping*. 2021;42(14):4685-4707. doi:10.1002/hbm.25578

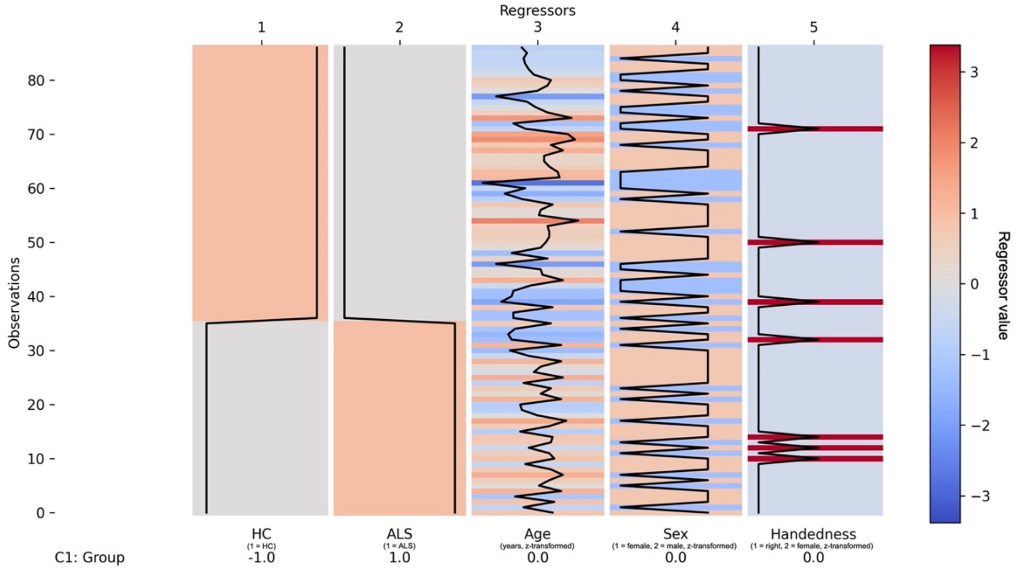

Supplementary Figure 1 – General linear model design – group comparison. Design matrix used to predict network metrics. The first regressor (HC) models the mean value of the network metric across healthy controls. The second regressor models the mean value of the network metric across amyotrophic lateral sclerosis (ALS) patients. The remaining regressors are included to model known sources of variability (age, sex, handedness) across participants. This has the effect of minimising the impact of these confounds on the group means. The confound regressors are calculated by z-transforming the values for age, sex (1=female or 2=male) and handedness (1=right 2=left) across participants.

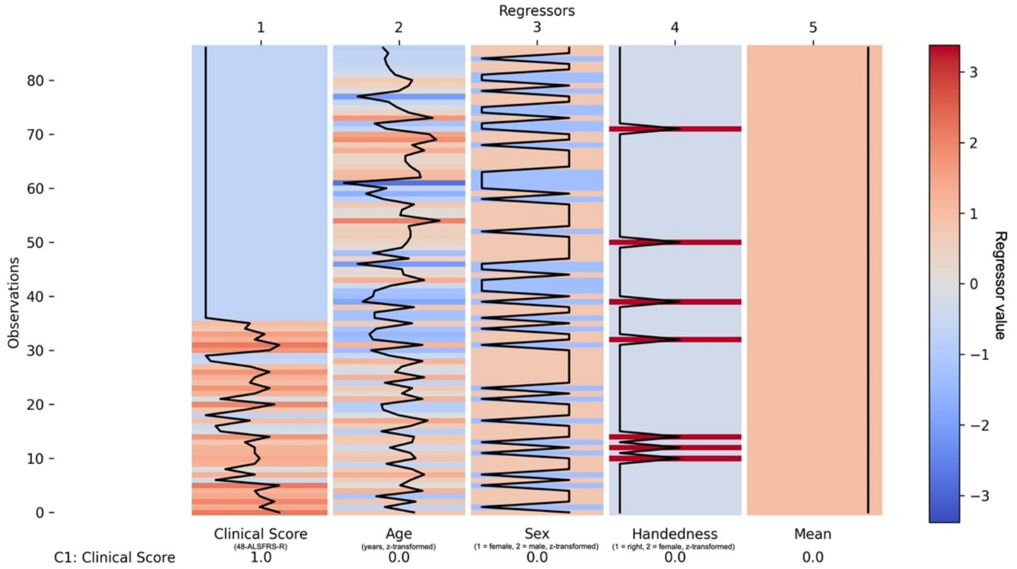

Supplementary Figure 2 - General linear model design – clinical score. Design matrix used to predict network metrics. The ‘Clinical Score’ regressor models the network metric across participants according to the clinical score. The ‘mean’ regressor models the mean value of the clinical scores across participants. The remaining regressors are included to model known sources of variability (age, sex, handedness) across participants. This has the effect of minimising the impact of these confounds on the group means. The confound regressors are calculated by z-transforming the values for age, sex (1=female or 2=male) and handedness (1=right 2=left) across participants.

Supplementary Table 1

Glasser52 parcellation co-ordinates. Parcel names and MNE-coordinates of parcel centres of the Glasser52 Parcellation.

Region Index	Location description	X	Y	Z
0	Primary and Early Visual Cortex Right	14.5	-80.5	-0.4
1	Dorsal Stream Visual Cortex Right	19.4	-81.4	30.6
2	Ventral Stream Visual Cortex Right	29.9	-57.6	-17.7
3	MT+ Complex and Neighbouring Visual Areas Right	42.2	-71.5	0.0
4	Superior Somatosensory and Motor Cortex Right	23.6	-29.5	61.0
5	Inferior Somatosensory and Motor Cortex Right	49.1	-13.4	38.3
6	Supplementary Motor Area Right	12.7	-2.2	62.9
7	Cingulate Motor Areas & Area 5 Right	10.0	-31.2	53.6
8	Premotor Cortex Right	40.9	0.9	41.1
9	Insular & Frontoparietal Operculum Right	37.7	1.6	4.0
10	Early Auditory Cortex Right	40.2	-27.5	13.5
11	Auditory Association Cortex Right	55.0	-14.0	-6.5
12	Medial Temporal Cortex Right	25.0	-22.1	-22.1
13	Lateral Temporal Cortex Right	48.5	-16.3	-25.3
14	Temporal-Parieto-Occipital Junction Right	52.1	-48.1	12.3
15	Medial Bank of the Intra-parietal Sulcus Right	29.5	-51.0	43.0
16	Superior Medial Parietal Cortex Right	19.1	-58.4	59.6
17	Inferior Parietal Cortex Task-Positive Network Right	56.7	-29.7	35.7
18	Inferior Parietal Cortex Task-Negative Network Right	47.4	-56.1	35.8
19	Intraparietal Sulcus & PGP Right	36.5	-71.1	32.6
20	Posterior Cingulate Cortex Right	10.3	-55.1	26.8

21	Anterior Cingulate and Medial Prefrontal Cortex Right	6.1	33.8	14.2
22	Orbital and Polar Frontal Cortex Right	17.1	47.0	-11.6
23	Inferior Frontal Cortex Right	45.4	32.5	3.8
24	Inferior Dorsolateral Prefrontal Cortex Right	34.8	37.1	25.7
25	Superior Dorsolateral Prefrontal Cortex Right	21.0	31.9	45.9
26	Primary and Early Visual Cortex Left	-16.9	-82.0	-0.9
27	Dorsal Stream Visual Cortex Left	-21.7	-84.3	27.0
28	Ventral Stream Visual Cortex Left	-34.5	-56.0	-18.6
29	MT+ Complex and Neighbouring Visual Areas Left	-45.2	-70.3	-1.4
30	Superior Somatosensory and Motor Cortex Left	-22.6	-30.4	62.8
31	Inferior Somatosensory and Motor Cortex Left	-48.3	-18.7	41.7
32	Supplementary Motor Area Left	-14.0	-2.7	62.7
33	Cingulate Motor Areas & Area 5 Left	-14.4	-30.6	49.7
34	Premotor Cortex Left	-40.3	-1.7	43.8
35	Insular & Frontoparietal Operculum Left	-41.3	0.1	3.7
36	Early Auditory Cortex Left	-45.4	-29.4	12.1
37	Auditory Association Cortex Left	-55.3	-17.3	-7.8
38	Medial Temporal Cortex Left	-27.9	-22.2	-22.6
39	Lateral Temporal Cortex Left	-49.6	-17.9	-25.1
40	Temporal-Parieto-Occipital Junction Left	-53.1	-54.0	14.5
41	Medial Bank of the Intra-parietal Sulcus Left	-31.1	-50.0	41.1
42	Superior Medial Parietal Cortex Left	-21.3	-60.5	58.2
43	Inferior Parietal Cortex Task-Positive Network Left	-56.6	-36.3	36.6
44	Inferior Parietal Cortex Task-Negative Network Left	-45.1	-63.4	35.2
45	Intraparietal Sulcus & PGP Left	-36.7	-70.0	30.4
46	Posterior Cingulate Cortex Left	-11.6	-49.5	28.0
47	Anterior Cingulate and Medial Prefrontal Cortex Left	-8.7	32.0	11.4
48	Orbital and Polar Frontal Cortex Left	-21.9	46.3	-8.1

49	Inferior Frontal Cortex Left	-45.1	28.2	7.7
50	Inferior Dorsolateral Prefrontal Cortex Left	-37.2	36.2	25.2
51	Superior Dorsolateral Prefrontal Cortex Left	-23.5	28.6	45.7

Supplementary Table 2

Full table of results. Summarises statistical analysis and results for each measure of cortical activity.

Analysis	Regressor	Frequency (0 = δ, 1 = θ, 2 = α, 3 = β, 4 = low-γ, 5 = high-γ)	Region Index (see **Supplementary Table 1**)	P-value	T-statistic	Degrees of Freedom	Betas
Power	Group	3	43	0.0346	3.7285	77	-0.0112046
		5	9	0.0152	3.99598	77	0.00105233
	UMNs	3	43	0.0376	3.75231	77	-0.0008812
		5	9	0.0248	3.86849	77	0.0002717
	ALSFRS-R	3	5	0.01	4.35708	77	-0.0011436
		3	8	0.031	3.95787	77	-0.0010335
		3	31	0.049	3.73635	77	-0.0010025
		3	43	0.036	3.88225	77	-0.000903
		5	8	0.0072	4.47261	77	0.00025741
		5	9	0.0112	4.31933	77	0.00029643
		5	35	0.0476	3.75132	77	0.00029307
	δALSFRS-R	-	-	-	-	-	-
Global AEC	Group	-	-	-	-	-	-
	UMNs	-	-	-	-	-	-
	ALSFRS-R	0	12	0.0256	3.87544	77	0.0193675
		1	0	0.0334	3.74061	77	0.0149693
		1	2	0.028	3.84553	77	0.018
		1	3	0.02	4.01486	77	0.0172175
		1	6	0.0382	3.6682	77	0.015254
		1	7	0.0496	3.53294	77	0.0150406
		1	12	0.046	3.56742	77	0.0168425
		1	14	0.0458	3.57154	77	0.0191558
		1	15	0.0452	3.5817	77	0.0167278
		1	25	0.008	4.39364	77	0.0174184
		1	26	0.0322	3.77014	77	0.0146173
		1	41	0.0384	3.65686	77	0.0175019
		1	42	0.0392	3.64757	77	0.0177192
		1	48	0.0366	3.69258	77	0.0145454
		1	49	0.0358	3.7047	77	0.0152475
		1	50	0.0032	4.72517	77	0.018696
		1	51	0.0206	3.99062	77	0.0161922
	δALSFRS-R	-	-	-	-	-	-

Intra-hemispheric AEC	ALSFRS-R	0	12	0.0176	4.11504	77	0.0213436
		1	12	0.046	3.66271	77	0.0186996
		1	25	0.0128	4.25499	77	0.0178713
		1	26	0.0356	3.79152	77	0.0147285
		1	27	0.0416	3.7084	77	0.0156078
		1	48	0.0372	3.77792	77	0.0154742
		1	50	0.0104	4.32431	77	0.0193207
		1	51	0.028	3.89821	77	0.0168422
		5	11	0.0418	3.70271	77	0.0218491
		5	13	0.0294	3.88149	77	0.0218211
		5	37	0.0322	3.83438	77	0.0208799
		5	43	0.0456	3.66459	77	0.0180593
	δALSFRS-R	-	-	-	-	-	-
Inter-hemispheric AEC	ALSFRS-R	1	2	0.0416	3.67856	77	0.0176203
		1	7	0.0382	3.73066	77	0.0178668
		1	15	0.035	3.76464	77	0.0190543
		1	16	0.0392	3.71367	77	0.0199032
		1	20	0.0456	3.61678	77	0.0167985
		1	24	0.005	4.69328	77	0.0180713
		1	25	0.0422	3.65718	77	0.0155422
		1	28	0.0174	4.10746	77	0.0203932
		1	29	0.012	4.32623	77	0.0183737
		1	30	0.031	3.83172	77	0.0164481
		1	40	0.0362	3.75685	77	0.0201482
		1	51	0.0228	3.96257	77	0.0169655
	δALSFRS-R	-	-	-	-	-	-
Inter:intra-hemispheric AEC	ALSFRS-R	1	24	0.0326	4.01843	77	0.0709009
		1	40	0.0066	4.52236	77	0.0702842
	δALSFRS-R	-	-	-	-	-	-
1/f exponent	Group	-	9	0.01	3.7822	77	-0.0633074
	UMNs	-	9	0.003	4.14987	77	-0.0633074
	ALSFRS-R	-	8	0.008	3.92443	77	-0.0531073
		-	9	0.002	4.2921	77	-0.0647963
	δALSFRS-R	-	9	0.024	3.53172	77	-0.0544932
